# HydroPrior: Offline LLM-Prior-Guided Residual Warning for Hydropower Bearing Vibration

**DOI:** 10.3390/s26206497

**Published:** 2026-10-14

**Authors:** Zile Dong, Daqing Zhong, Yanru Zhang

**Affiliations:** 1 Shenzhen Institute for Advanced Study, University of Electronic Science and Technology of China, Shenzhen 518110, China; ziledong@uestc.edu.cn (Z.D.); zhongdaqing@alu.uestc.edu.cn (D.Z.); 2 School of Computer Science and Engineering, University of Electronic Science and Technology of China, Chengdu 611731, China

**Keywords:** hydropower vibration monitoring, residual warning, proxy warning, LLM priors, lightweight forecasting, condition monitoring, Balanced Warning Score

## Abstract

**Highlights:**

**What are the main findings?**

- Frozen LLM-derived operating priors improve multi-horizon lightweight vibration forecasting on the evaluated hydropower record.
- HydroPrior achieves a BWS of 0.7492 under a fixed proxy-warning protocol and remains ahead in equal-budget comparisons.

**What are the implications of the main findings?**

- Offline semantic priors can enrich sensor analytics while keeping routine execution lightweight and deterministic.
- Joint reporting of transition coverage and alert burden supports transparent screening in label-scarce condition monitoring.

**Abstract:**

Hydropower vibration monitoring requires early warnings with manageable inspection costs despite scarce verified fault labels. HydroPrior combines offline large language model (LLM) priors with lightweight residual-based warning. The LLM converts channel descriptions and training-set statistics into frozen operating-context features, which enter a compact forecasting correction head. Residual scoring, calibrated thresholds, and contextual confirmation then produce budgeted alerts. Evaluation uses rapid vibration transitions as proxy events and the Balanced Warning Score (BWS) to summarize event coverage and alarm burden. On a public hydropower bearing-vibration dataset, HydroPrior obtains the lowest average forecasting error among the evaluated models. Under the proxy-warning protocol, its mean BWS is 0.7492 (standard deviation: 0.0405), with a transition-hit rate of 0.9106, 0.15 false alarms per 1000 non-transition points, and 0.0199 alert coverage. Comparisons using a common confirmation mechanism and coverage budget also favor HydroPrior. The findings support the feasibility of frozen operating priors for lightweight vibration forecasting and focused warning on the evaluated unit record.

## 1. Introduction

Hydropower units are high-value rotating assets whose bearings support continuous operation under changing load, water head, flow, and dispatch schedules. A developing bearing problem can increase vibration, accelerate component wear, and eventually force an unplanned shutdown. Vibration monitoring is therefore an important source of early operational evidence because it reflects mechanical, hydraulic, electromagnetic, and degradation-related effects [1,2]. A useful plant warning system must detect meaningful changes early, keep the number of alerts within an inspectable range, and explain each alert through an observable operating context.

These requirements are difficult to satisfy with plant data. Operating records and fault histories are commercially and operationally sensitive, which limits the availability of verified point-level fault labels. Maintenance logs are also much coarser than high-frequency sensor streams. At the same time, routine changes in load and hydraulic conditions can alter vibration in ways that resemble early abnormal behavior. The resulting task is best treated as label-scarce anomaly warning: the model must recognize unusual signal behavior while controlling the inspection burden created by false or persistent alerts.

Traditional time-series models offer transparent and inexpensive monitoring tools. Persistence, exponential smoothing, and autoregressive predictors are easy to maintain and provide computationally inexpensive reference methods [3,4]. Their fixed structures capture regular temporal behavior well, while complex hydraulic–mechanical coupling and nonlinear interactions across multiple channels require richer representations.

Data-driven forecasting models capture temporal structure through several complementary architectures. Long short-term memory (LSTM) recurrent networks model state evolution, decomposition-linear models separate trend and residual behavior, patch-based models encode local temporal structures, and Transformer-family models capture broader dependencies [5,6,7,8,9,10,11,12]. They provide strong residual generators for forecasting-based warning. Their learned representations remain tied to the available training data, so sparse labels and limited operating coverage can leave important engineering relations, such as load tendency, thermal response, and cross-bearing vibration coupling, only partially represented.

Recent time-series foundation models and large language model (LLM)-based forecasting methods provide another source of knowledge. They reprogram language models for numerical sequences, represent time series as tokens, or learn transferable priors from heterogeneous collections [13,14,15]. Their pretrained representations motivate the use of reusable temporal knowledge; LLMs also offer an interface for organizing channel descriptions and operating relations. Continuous plant monitoring calls for predictable latency, modest hardware requirements, deterministic execution, and traceable rules. These requirements favor a lightweight routine warning path.

HydroPrior combines the complementary strengths of LLMs and lightweight models [16]. The LLM is used once, before model training, to turn channel descriptions and training-set statistics into a frozen set of operating priors. A lightweight model uses these numerical priors for forecasting and warning. This division of work brings engineering context into the model while retaining a compact, deterministic inference path. It also makes the LLM contribution inspectable because every prior feature is linked to a named rule and a training-set statistic.

The study uses the Bearing Vibration Dataset of a Hydropower Project, which contains operating, temperature, and guide-bearing vibration measurements from a real unit [17]. Previous work has used the same source for condition monitoring and prognostics [18]. HydroPrior predicts upper-guide-bearing vertical vibration, converts forecast residuals into warning scores, calibrates a score threshold for each prediction horizon, and confirms alerts using non-target operating and vibration context. Rapid vibration transitions provide reproducible proxy events for evaluating the warning stream when verified fault labels are unavailable.

The investigation is organized around three research questions. *RQ1* asks whether frozen LLM-derived operating priors improve forecasting under the common data and training protocol. *RQ2* asks whether HydroPrior improves rapid-transition proxy-event coverage and false-alarm control when compared models receive the same alert-coverage budget. *RQ3* asks whether this knowledge-enhanced design preserves a lightweight execution path and a traceable mapping from operating rules to numerical prior features. These questions link the method components directly to the forecasting, equal-budget warning, and reproducibility evidence reported in Section 4.

This work makes four contributions. First, it defines a reproducible forecasting-to-warning protocol for label-scarce hydropower monitoring, including fixed data splits, preprocessing, forecasting horizons, prior generation, and warning evaluation. Second, it proposes HydroPrior, which converts offline LLM-derived operating knowledge into five frozen prior features and injects them through a bounded correction head attached to a lightweight forecaster. Third, it introduces the Balanced Warning Score (BWS) to measure rapid-transition coverage, false-alarm density, and alert coverage under pre-specified operator-inspection budgets. Fourth, it evaluates HydroPrior against statistical, recurrent, decomposition-linear, patch-based, and Transformer-family models under the same forecasting and warning settings. HydroPrior achieves the lowest average forecasting error and a BWS of 0.7492±0.0405 under the proposed proxy-warning protocol.

The remainder of the paper reviews the relevant forecasting and anomaly-warning literature, presents HydroPrior and the common warning protocol, reports forecasting and warning results, and concludes with practical implications and the scope of the current evidence.

## 2. Related Work

### 2.1. Deep Time-Series Forecasting Backbones

Neural forecasting first developed around recurrent models such as LSTM, whose gated memory captures long temporal dependencies [5]. Transformer architectures [6] later enabled long-sequence models, including Informer [7], Autoformer [8], and Non-stationary Transformers [19]. These methods address attention cost, temporal decomposition, and distribution shift. Simpler structures remain highly competitive: DLinear combines decomposition with linear mappings [9], while PatchTST represents local segments as tokens [10]. TimesNet [11], iTransformer [12], and TimeMixer [20] extend the field through two-dimensional temporal variation, inverted tokenization, and multiscale mixing.

Forecasting-based warning depends on both prediction accuracy and the shape of the resulting residual stream. Recurrent memory favors short-term adaptation, patch-based models capture local motifs, and decomposition-linear mappings separate slow and fast components before prediction. These choices affect residual magnitude, tail behavior, burstiness, and smoothness. We use the term *residual calibratability* for the ability to convert these errors into a stable alert stream under a common threshold and alert budget. This view provides a consistent way to compare heterogeneous forecasters as upstream components of the same warning system.

### 2.2. Unsupervised Time-Series Anomaly Detection

Unsupervised anomaly detection often uses reconstruction error, prediction error, or representation discrepancy. Early neural approaches used LSTM encoder–decoder prediction and reconstruction errors for time-series anomalies [21]. GDN uses learned sensor dependency graphs for multivariate anomaly detection [22]. OmniAnomaly models stochastic recurrent dynamics for multivariate anomaly detection [23], USAD uses adversarially trained autoencoders [24], and TranAD applies transformer architectures to multivariate anomaly detection [25]. Anomaly Transformer introduces association discrepancy to separate normal and abnormal temporal associations [26]. LSTM-based prediction with dynamic thresholding has also been used for operational anomaly detection in spacecraft telemetry [27].

Prediction-error methods suit label-scarce plants because they learn temporal regularities in the training record and flag departures from the forecast. Turning a prediction error into a warning requires score construction, threshold calibration, temporal processing, and an explicit alarm budget. Reviews show that these choices can substantially affect reported performance [28,29,30]. Public benchmarks commonly emphasize point-level scores or adjusted F1, whereas plant operation also requires event coverage and false-alarm control. Our protocol makes this residual-to-warning conversion explicit and evaluates rapid-change proxy events under fixed budgets.

Industrial vibration monitoring places particular weight on false-alarm control. Heavy-industry predictive-maintenance studies identify false-alarm reduction as a central deployment objective [31], and structural-health monitoring under variable operating conditions uses condition-dependent alarm levels [32]. Hydropower and rotating-machinery studies also interpret vibration together with machine state, maintenance knowledge, and operator-reviewed intervals [33,34]. These studies motivate joint consideration of machine state and alarm burden. In the present evaluation, rapid-change events provide a reproducible signal-based proxy; maintenance records and expert-reviewed windows would provide additional evidence for fault attribution.

Benchmark reliability is a related concern. Weak labels and permissive evaluation rules can create an illusion of progress in time-series anomaly detection [35]. The hydropower data used here contain operating signals without verified point-level fault labels. We therefore report rapid-change proxy-warning evidence and state this scope wherever warning results are interpreted.

Threshold calibration converts a score distribution into an alert stream. The hit rate then measures the coverage of rapid-change proxy events under that rule. The calibration procedure and evaluation scope are specified in the following sections.

### 2.3. LLM-Prior-Guided Lightweight Modeling for Time Series

Large language models and time-series foundation models have broadened sequence modeling. LLMTime performs zero-shot forecasting by representing numerical sequences as text-like tokens [13]; Time-LLM aligns time-series patches with textual prototypes [14]; and Chronos trains probabilistic token models across heterogeneous series [15]. Together, these studies show that pretrained models can supply reusable temporal and semantic priors. Hydropower warning adds strict requirements for latency, deterministic execution, and traceability, which favor using the LLM before deployment and retaining a lightweight model for routine prediction and warning.

HydroPrior follows this division of work. An offline LLM converts channel semantics and training-set statistics into named operating priors, and a lightweight model handles forecasting and warning. Features describing load, thermal behavior, vibration coupling, and operating change can be traced to their source rules. Matched comparisons with no-prior, statistical-feature, and learned-feature controls evaluate the contribution of the prior-generation strategy. Operator review would further establish whether the rule descriptions provide useful maintenance context.

### 2.4. Industrial Vibration Monitoring and Deployment Constraints

Industrial vibration monitoring must accommodate continuous acquisition, changing operating regimes, maintenance lead times, and the cost of missed or false alarms. Prognostics and predictive-maintenance surveys therefore connect model outputs with maintenance value, alarm burden, and continuous-use feasibility [36,37,38]. Rolling-bearing studies also show that vibration signatures vary with operating condition, preprocessing, and degradation stage [39,40]. For hydropower warning, these findings translate into a practical requirement: alerts should cover important changes while remaining sparse enough for routine inspection.

BWS implements this requirement by combining rapid-change coverage, false-alarm density, and alert coverage. The metric evaluates warning behavior under the current proxy-event protocol; maintenance-log studies are needed for fault-level validation.

## 3. Materials and Methods

**Figure 1:**
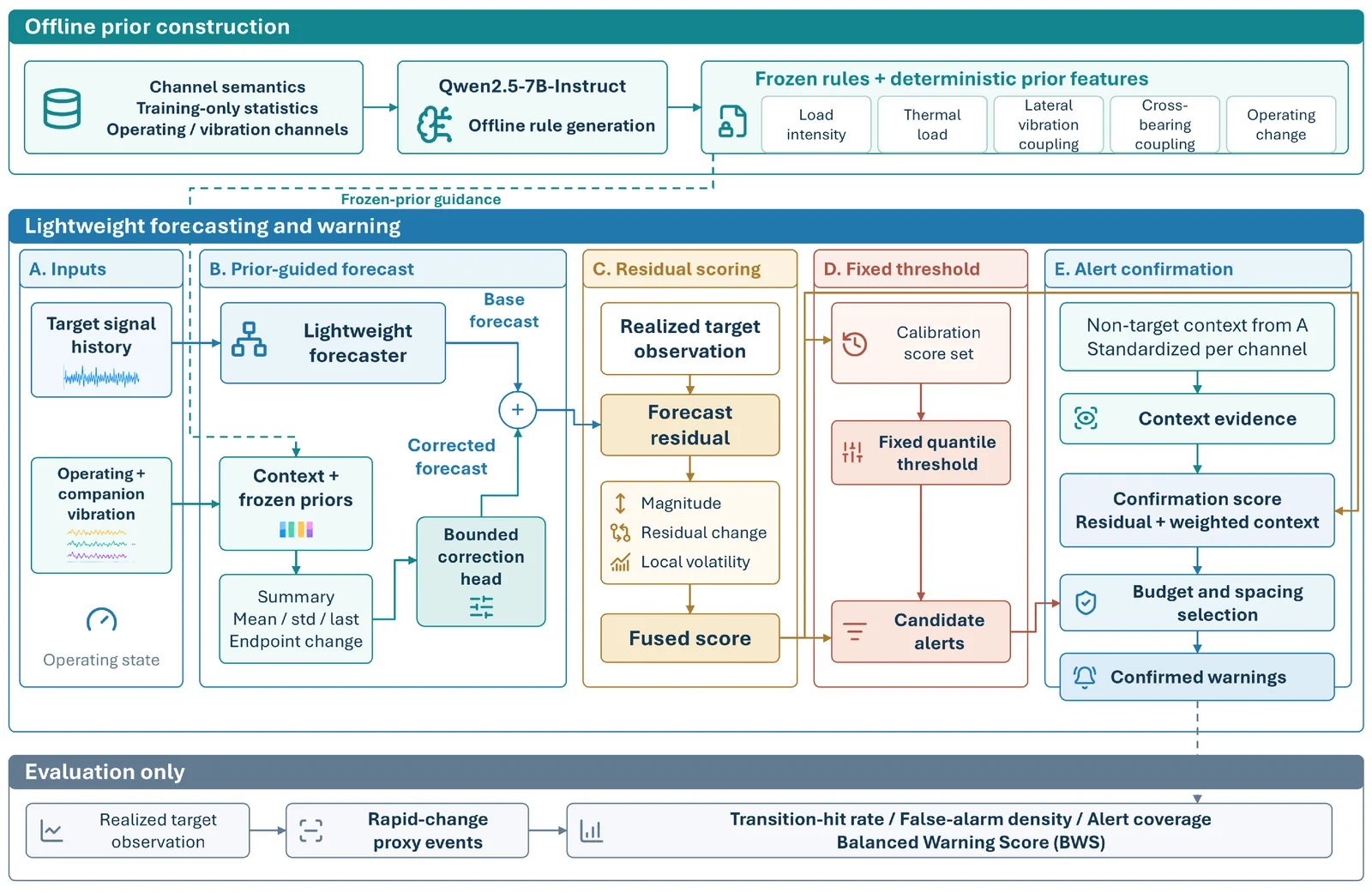
Overview of the proposed forecasting-to-warning method. Sensor streams are mapped to forecasts, residuals are fused into warning scores, a calibration-window threshold produces candidate alerts, and HydroPrior adds an adaptive alert-confirmation layer before the Balanced Warning Score (BWS) summarizes proxy-warning evidence. Offline large language model (LLM) priors provide traceable operating-context features through a lightweight residual-correction head. Repeated target-observation and non-target-context labels refer to the same respective data streams. Solid arrows show numerical data flow; teal dashed arrows show frozen-prior guidance, and gray dashed arrows connect the fixed warning stream to evaluation. The signal sketches and feature bars are schematic illustrations.

OpenAI Codex assisted with manuscript drafting and language revision, and GPT Image 2 provided preliminary layout references. Figure 1 was redrawn as an editable PowerPoint diagram with cropped AI-generated schematic icons and checked against the method definitions. These preparation tools are distinct from Qwen2.5-7B-Instruct, the offline prior-generation component described below. The authors reviewed and edited the resulting text and artwork.

### 3.1. Forecasting-to-Warning Formulation

HydroPrior separates signal prediction from warning decisions. A forecasting model maps a fixed historical window to a multi-horizon prediction. The observed prediction errors then pass through a common score and threshold interface. Proxy-event labels are used only for evaluation and never enter backbone training or alert generation.

The method can be viewed as a five-stage pipeline:

(1) $$D_{\mathrm{proxy}}=E_{\mathrm{BWS}}\circ C_{\kappa ,\epsilon ,\Delta_{\mathrm{alert}}}\circ T_{q,W_{\tau }}\circ S_{a,b,c,w}\circ F_{\theta },$$

Here, $F_{\theta }$ produces forecasts, $S_{a,b,c,w}$ converts residuals into scalar warning scores, and $T_{q,W_{\tau }}$ applies the calibration-window quantile threshold. The operator $C_{\kappa ,\epsilon ,\Delta_{\mathrm{alert}}}$ confirms candidate alerts subject to coverage, audit-tail, and spacing constants, and $E_{\mathrm{BWS}}$ evaluates the fixed alert stream. Every model uses the same score operator. For residual-only baselines in the raw comparison, C is the identity; the equal-budget comparison applies the same confirmation operator to all included forecasters.

Table 1 fixes the time and intermediate-variable notation used throughout the method.

Let $y_{i}$ denote the target vibration signal at realized timestamp *i*, let *t* be the forecast origin, let $x_{t-L+1:t}$ denote the length-*L* input window, and let $f_{\theta }$ be a forecasting backbone. Given $x_{t-L+1:t}$, the model predicts a horizon-*H* trajectory

(2) $${\hat{y}}_{t+1:t+H}=f_{\theta }\left(x_{t-L+1:t}\right).$$

For each horizon *h*, one forecast is aligned with each realized test value at i=t+h. The aligned forecast is

(3) $${\hat{y}}_{i|t}^{\left(h\right)}={\left[f_{\theta }\;\left(x_{t-L+1:t}\right)\right]}_{h},\;i=t+h\in I_{\mathrm{test}}^{\left(h\right)},$$

where ${\left[\cdot \right]}_{h}$ selects the *h*-th forecast value and $I_{\mathrm{test}}^{\left(h\right)}$ contains test timestamps with enough history. This alignment yields one residual per timestamp and avoids averaging forecasts issued at different times. The aligned residual is

(4) $$e_{i|t}=y_{i}-{\hat{y}}_{i|t},\;r_{i|t}=\left|e_{i|t}\right|.$$

Here $e_{i|t}$ retains the direction of the forecasting error, and $r_{i|t}$ is the non-negative residual magnitude used by the warning score. For compactness, downstream equations write the horizon-aligned sequence as $e_{i}$ and $r_{i}$. The warning score combines residual magnitude, residual change, and local residual volatility:

(5) $$s_{i}=ar_{i}+b\left|r_{i}-r_{i-1}\right|+c\;\mathrm{std}\left(r_{i-w+1:i}\right).$$

The coefficients *a*, *b*, and *c* control the relative emphasis on residual level, residual change, and local residual volatility, while *w* controls the local volatility window. Direct thresholding of $r_{i}$ provides a residual-based warning criterion. The fused score retains this instantaneous mismatch and adds temporal context: $|r_{i}-r_{i-1}|$ measures the magnitude of change between successive residual magnitudes, and the standard-deviation term describes fluctuations around the local mean residual magnitude. All three terms are derived from the same forecast error and jointly describe its current level, rapid changes, and local variability.

The weighted sum provides an inspectable score that is thresholded in the same way for every forecaster.

Load, water head, flow, and dispatch schedules influence vibration and forecast-residual distributions. A calibration score set provides the reference distribution for thresholding. Let $C^{\left(h\right)}$ be the set of calibration-score indices at horizon *h*, and let $W_{\tau }^{\left(h\right)}=\left|C^{\left(h\right)}\right|$ denote its size. With $Q_{q}\left(\cdot \right)$ denoting the empirical quantile at probability *q*, the threshold is

(6) $$\tau^{\left(h\right)}=Q_{q}\left(\{s_{j}^{\left(h\right)}|j\in C^{\left(h\right)}\}\right).$$

The calibrated threshold remains fixed during warning generation. The experimental settings specify the calibration-set construction and evaluation population. Each score is converted into a binary alert indicator by

(7) $$\alpha_{i}=\left\{\begin{array}{cc} 1, & s_{i}^{\left(h\right)}>\tau^{\left(h\right)},\\ 0, & s_{i}^{\left(h\right)}\leq \tau^{\left(h\right)}, \end{array}\right.$$

Here, $\alpha_{i}=1$ marks a candidate warning and $\alpha_{i}=0$ leaves the point unflagged. A larger *q* selects a smaller upper tail of the calibration score distribution and therefore produces a tighter alert stream. HydroPrior passes these candidate alerts to the confirmation layer defined below.

The Experimental Section fixes the numerical weights in (5) and keeps them shared across compared models.

### 3.2. Generic Forecasting Backbone Interface

The forecasting component uses a common interface that accepts different model families. Let $m\in M_{\exp}$ identify a forecaster. Each model maps the same input window to an *H*-step prediction:

(8) $${\hat{y}}_{t+1:t+H}^{\left(m\right)}=f_{\theta_{m}}^{\left(m\right)}\left(x_{t-L+1:t}\right),$$

and the base forecasting objective for trainable models is

(9) $$L_{\mathrm{pred}}^{\left(m\right)}=\frac{1}{|W_{\mathrm{train}}|H}\sum_{t\in W_{\mathrm{train}}}\sum_{h=1}^{H}\ell \left(y_{t+h},{\hat{y}}_{t+h}^{\left(m\right)}\right).$$

where $W_{\mathrm{train}}$ is the set of training windows and ℓ(·,·) is the forecasting loss. The residual-warning layer is independent of the internal structure of $f_{\theta_{m}}^{\left(m\right)}$; it only consumes the signed error stream

(10) $$e_{i|t}^{\left(m\right)}=y_{i}-{\hat{y}}_{i|t}^{\left(m\right)},\;i=t+h,$$

and its magnitude $r_{i|t}^{\left(m\right)}=\left|e_{i|t}^{\left(m\right)}\right|$ before applying the score definition in (5). This common interface connects each forecaster to residual scoring and alert conversion. The experimental settings specify the backbone configurations used in the comparison.

The common interface gives every model the same prediction target, residual definition, score function, and threshold rule. It also allows the prior mechanism to be attached as a bounded correction path without changing the forecasting task.

Numerical histories alone provide limited access to the engineering meaning of load changes, thermal trends, cross-channel vibration coupling, and operating transitions. HydroPrior adds this context through frozen numerical priors generated by an offline LLM and a small residual-correction head.

### 3.3. Offline LLM Priors and Small-Model Residual Correction

HydroPrior combines a lightweight forecaster, frozen LLM-prior features, a bounded correction head, and contextual alert confirmation. Qwen2.5-7B-Instruct generates the rules during offline prior construction. The rules are then frozen, and routine online execution uses numerical features, a DLinear backbone, a two-layer MLP correction head, and deterministic alert confirmation. Here, lightweight refers specifically to this compact online forecasting-and-warning path. Computationally heavier LLM inference is confined to offline preparation. The correction head and confirmation procedure add computation relative to standalone DLinear to enable operating-context correction and controlled warning generation. This definition concerns the routine execution path; the complete offline-to-online pipeline also includes the separate cost of LLM prior construction. The reported instance uses five prior features derived from multichannel operating data. Forecasting metrics are computed from the prior-corrected prediction before alert confirmation, and warning metrics are computed from the confirmed alert stream.

The LLM is invoked once before model training. It receives channel names, physical descriptions, and training-set statistics, and returns rules for channel grouping and operating cues such as load intensity, thermal tendency, vibration coupling, and operating change. After validation, the rule file is frozen and reused unchanged throughout data preparation, model training, and warning evaluation. Deterministic scripts convert the rules into numerical features using training-set normalization constants and quantiles.

Reproducibility is anchored in the frozen artifact: the prompt, model identifier, decoding controls, validated rules, and transformation constants are recorded before fitting the forecaster. Section 4 lists the concrete settings.

Formally, the frozen rule file is written as

(11) $$R={\left\{\left(G_{k},\omega_{k},\psi_{k}\right)\right\}}_{k=1}^{K},$$

where $G_{k}$ is a channel group, $\omega_{k}$ is a rule weight or direction, and $\psi_{k}$ is a deterministic transformation such as normalized level, cross-channel contrast, or high-quantile transition tendency. The rule file is generated once and then treated as fixed metadata. This makes the LLM contribution auditable: the model supplies structured operating priors, while all numerical values used during training and evaluation are computed by deterministic scripts.

Let $x_{t}=\left[u_{t},y_{t}\right]$ contain exogenous channels $u_{t}$ and the target vibration channel $y_{t}$. The LLM-prior feature generator produces

(12) $$p_{t}=\phi_{\mathrm{LLM}}\left(u_{1:t};R,S_{\mathrm{train}}\right),$$

where R is the frozen rule set and $S_{\mathrm{train}}$ contains training-segment scaling and quantile statistics. The augmented input is

(13) $${\tilde{x}}_{t}=\left[u_{t},p_{t},y_{t}\right].$$

Each prior feature can therefore be traced to a named rule and a training-set statistic. The five-dimensional prior vector describes load intensity, thermal load, upper-guide-bearing lateral vibration coupling, cross-bearing vibration coupling, and operating change. Appendix A lists the corresponding channel groups, transformations, weights, and statistics.

All normalization constants, quantiles, and clipping ranges are fitted on the training segment and then fixed for validation and testing. The LLM supplies the semantic rule structure; observed sensor values determine the time-varying feature values.

The prior interface is a residual-correction module that can be attached to any forecasting backbone in the experimental set $M_{\exp}$. A base model first produces a target-channel forecast

(14) $${\hat{y}}^{\left(0,m\right)}=f_{\theta_{m}}^{\left(m\right)}\left(y_{t-L+1:t}\right).$$

Before aggregation, every non-target channel *c* is standardized independently with its training-segment median $m_{c}$ and interquartile range $I_{c}$:

(15) $${\tilde{u}}_{j,c}=\frac{u_{j,c}-m_{c}}{\max(I_{c},\varepsilon_{s})}.$$

Here, $\varepsilon_{s}$ prevents division by zero for constant channels, and values are clipped to their training-derived robust ranges. Let $v_{t}=\left[{\tilde{u}}_{t},{\tilde{p}}_{t}\right]$ concatenate the standardized non-target channels and prior features, with all scaling statistics fitted on the training split. Their input-window mean, standard deviation, last value, and endpoint difference form

(16) $$h_{\mathrm{ctx}}=\left[\mu \left(v_{t-L+1:t}\right),\sigma \left(v_{t-L+1:t}\right),v_{t},v_{t}-v_{t-L+1}\right].$$

A lightweight multilayer perceptron (MLP) $g_{\psi }$ then produces the explicitly bounded correction

(17) $$\begin{array}{c} \Delta {\hat{y}}^{\left(m\right)}=0.4\sigma_{y}\tanh\;\left(g_{\psi }\left(h_{\mathrm{ctx}}\right)\right),\\ {\hat{y}}^{\left(m,+\right)}={\hat{y}}^{\left(0,m\right)}+\Delta {\hat{y}}^{\left(m\right)}. \end{array}$$

where $\sigma_{y}$ is the target standard deviation fitted on the training segment. Consequently, every output coordinate satisfies $|\Delta \hat{y}|\leq 0.4\sigma_{y}$. The correction head is zero-initialized so that training starts from the base forecast and introduces context only through the forecasting objective. Its width, activation, and dropout are specified once in the experimental settings.

The prior branch translates operating relationships into numerical context for forecast correction. Load variation, thermal conditions, and companion-bearing vibration provide context for interpreting changes in the target vibration signal. Frozen rules convert these observations into prior features, which are summarized over the input window and passed to the correction head. Through the forecasting objective, the correction head learns how this context should adjust the base forecast. For an additive correction $\Delta {\hat{y}}_{i}$, the corrected residual satisfies

(18) $$e_{i}=y_{i}-\left({\hat{y}}_{i}^{\mathrm{base}}+\Delta {\hat{y}}_{i}\right)=e_{i}^{\mathrm{base}}-\Delta {\hat{y}}_{i},\;r_{i}=\left|e_{i}\right|.$$

Here, ${\hat{y}}_{i}^{\mathrm{base}}$ and $e_{i}^{\mathrm{base}}$ denote the base forecast and its signed error. The warning score uses the residual magnitude $r_{i}$. The context-dependent correction therefore influences both residual magnitude and temporal variation before warning-score construction. The correction head learns the magnitude and direction of this adjustment through the forecasting objective. Matched forecasting and warning comparisons assess whether this contextual adjustment improves prediction accuracy and warning performance.

### 3.4. Adaptive Alert Confirmation Layer

All models use the residual score in (5) and the calibration-window threshold in (6) to produce candidate alerts. HydroPrior then applies an adaptive confirmation layer. This layer ranks candidate alerts with short-term evidence from operating and companion-vibration channels, while leaving the forecasting model and common residual score unchanged.

Let $\alpha_{i}^{0}$ be the candidate alert. HydroPrior constructs an auxiliary confirmation evidence term from operating-context and companion-vibration changes:

(19) $$c_{i}=\text{robust\_scale}\left({\mathrm{median}}_{c\in X_{\mathrm{ctx}}}\left|\Delta {\tilde{x}}_{i,c}\right|\right),$$

where $X_{\mathrm{ctx}}$ contains operating and companion-vibration channels, ${\tilde{x}}_{i,c}$ is independently standardized by (15), and robust_scale(·) uses calibration statistics after cross-channel aggregation. The term $c_{i}$ is large when a residual-based candidate coincides with coherent changes in load, speed, temperature, or cross-bearing vibration. HydroPrior combines this evidence with the residual score:

(20) $$z_{i}={\bar{s}}_{i}+\lambda_{c}{\bar{c}}_{i},$$

where ${\bar{s}}_{i}$ and ${\bar{c}}_{i}$ are normalized with calibration-set minima and maxima; evaluation values are clipped to that range. The coefficient $\lambda_{c}$ controls the contribution of operating-context evidence. A deterministic budget operator then selects the confirmed alerts:

(21) $$\alpha^{\mathrm{HP}}=C_{\kappa ,\epsilon ,\Delta_{\mathrm{alert}}}\left(\alpha^{0},z\right),$$

Here, κ is the alert-coverage budget, ϵ is an audit-tail allowance, and $\Delta_{\mathrm{alert}}$ is the minimum alert spacing. Within each timestamp-contiguous segment, candidates are processed in descending $z_{i}$ order and retained when their distance from every already retained alert is at least $\Delta_{\mathrm{alert}}$ samples, subject to the coverage budget. A timestamp gap starts a new segment. Transition labels are introduced afterward when BWS is computed. Section 4 gives the fixed values of $\lambda_{c}$, κ, ϵ, and $\Delta_{\mathrm{alert}}$.

### 3.5. Balanced Warning Score

BWS summarizes the alert stream $\left\{\alpha_{i}\right\}$ under a common deployment budget. It rewards rapid-change coverage and compliance with the specified false-alarm and alert-coverage budgets.

Transition events are proxy events generated from rapid signal changes. Let

(22) $$\delta_{y}=Q_{0.95}\left(\left\{\right|y_{i}-y_{i-1}{\left|\right\}}_{i}\right)$$

be the empirical 95th percentile of absolute first differences over the evaluated sequence. The transition proxy is

(23) $$v_{i}=I\left[|y_{i}-y_{i-1}|>\delta_{y}\right].$$

A transition is counted as hit if any alert $\alpha_{i}=1$ appears in a local tolerance window around an index with $v_{i}=1$. Let $r_{\mathrm{hit}}$ be the transition-hit rate, *f* be the false alarms per 1000 non-transition points, and ρ be the alert coverage. BWS uses fixed deployment budgets $f_{0}$ and $\rho_{0}$:

(24) $$\begin{array}{cc} \mathrm{BWS}= & 0.45r_{\mathrm{hit}}+0.35\left(1-\min\left(\frac{f}{f_{0}},1\right)\right)\\ \; & +0.20\left(1-\min\left(\frac{\rho }{\rho_{0}},1\right)\right). \end{array}$$

The weights give the greatest importance to rapid-change coverage, followed by false-alarm-budget and alert-coverage-budget compliance. Below a budget, a smaller burden receives a larger contribution. Once $f\geq f_{0}$ or $\rho \geq \rho_{0}$, the corresponding contribution is clipped to zero; further excess leaves that contribution at zero. Because the budgets are fixed, a model’s BWS remains unchanged when another baseline is added or removed.

Algorithms 1 and 2 separate alert generation from proxy evaluation. Alerts are fixed before rapid-transition labels are used to calculate the metrics.
**Algorithm 1:** Residual-warning alert generation._1_Train the forecasting model using the data split defined in the experimental settings and generate evaluation forecasts._2_For each horizon *h*, align the forecast issued at t=i−h with $y_{i}$ using (3); compute one residual per realized timestamp within each horizon–seed stream._3_Compute $s_{i}$ using residual magnitude, residual change, and rolling residual volatility._4_Estimate the horizon-specific threshold $\tau^{\left(h\right)}$ from the specified calibration score set._5_Generate candidate alerts $\alpha_{i}^{0}=I\left[s_{i}^{\left(h\right)}>\tau^{\left(h\right)}\right]$._6_Apply $C_{\kappa ,\epsilon ,\Delta_{\mathrm{alert}}}$ when confirmation is enabled; otherwise, retain the candidate stream.

**Algorithm 2:** Offline proxy-warning evaluation.


_1_
Construct rapid-transition proxy events from $v_{i}=I[|y_{i}-y_{i-1}\left|>\delta_{y}\right]$.
_2_
Count a proxy event at *j* as hit if an alert occurs at *i* with |i−j|≤6; truncate the window at sequence boundaries.
_3_
Compute false alarms per 1000 non-transition points and alert coverage.
_4_
Compute BWS using the fixed budgets $f_{0}$ and $\rho_{0}$.
_5_
Report BWS together with hit, false-alarm density, and alert coverage.



This ordering prevents proxy labels from influencing alert generation and makes each evaluation step reproducible.

## 4. Results and Discussion

### 4.1. Experimental Settings

The implementation environment uses Python 3.11.15 and PyTorch 2.11.0 with CUDA 12.8 support on NVIDIA GeForce RTX 4090 hardware (NVIDIA Corporation, Santa Clara, CA, USA). Data processing uses NumPy 2.1.2, pandas 2.3.3, SciPy 1.16.3, and scikit-learn 1.7.2. Public release of the custom implementation has not been authorized; the code is currently unavailable for public distribution.

All comparisons use the data splits, input length, forecast horizons, training schedule, prior-generation procedure, residual-score weights, and warning settings specified below. Forecasting parameters and channel-normalization statistics are fitted on the training split. The validation split supports model selection and warning-threshold calibration. Thresholds are estimated from validation scores and held fixed during test evaluation.

The input length L=96 supplies 16 h of history at the 10 min sampling interval. This provides multi-hour vibration and operating context with a compact input representation and is twice the longest forecast horizon, H=48 (eight hours). The same L=96 window is used for all forecasting models to ensure a controlled comparison. Establishing an optimal history length requires a dedicated window-length study.

The residual-score coefficients a=0.5, b=0.3, and c=0.2 are fixed design coefficients shared across all compared models. Residual magnitude receives the largest coefficient because the current prediction mismatch is the primary warning signal. Residual change receives 0.3 to represent temporal change in that mismatch, and local residual volatility receives 0.2 to provide short-window fluctuation context. These coefficients were fixed before final test evaluation; they were neither optimized against the test set nor selected by test-set performance. They specify the emphasis adopted for this score design, with no claim of unique optimality, and are distinct from the BWS evaluation weights. The score threshold is calibrated using validation data and held fixed during test evaluation. Direct absolute-residual thresholding is the direct residual-based alternative. The fused score is proposed as a temporally informed extension of residual thresholding, with all components derived from the forecast error. The reported evaluation focuses on the complete warning formulation under the common protocol.

The six-sample alert spacing separates retained alert-positive records by at least one hour within regular 10 min segments, reducing closely clustered records and making the coverage budget easier to interpret for inspection. Candidates are processed in descending confirmation-score order and retained when they satisfy this spacing from already retained records. The spacing is an alert-management design choice, with a new segment beginning after a timestamp gap.

For proxy-event evaluation, the tolerance radius is six evaluation positions: an event at *j* is counted as hit when at least one alert-positive record occurs at *i*, satisfying |i−j|≤6. The full window contains up to 13 positions and is truncated at sequence boundaries. For consecutive 10 min observations, it extends 60 min before and 60 min after the event. This fixed convention associates nearby alerts with a local rapid-change neighborhood and is shared across models. It measures temporal proximity to proxy events; the symmetric window does not establish an early-warning lead time. The tolerance window and alert spacing serve separate evaluation and selection roles.

The experiment uses the Bearing Vibration Dataset of a Hydropower Project [17]. It contains power output, unit speed, bearing-pad temperatures, and vibration measurements from the upper, lower, and turbine guide bearings. Upper-guide-bearing vertical vibration is the prediction and warning target because it directly measures vertical rotor–bearing motion. The remaining channels supply operating context to the multichannel model and prior generator. Forecast horizons are 1, 12, 24, and 48. Every model uses the same chronological split, normalization, input window, horizons, residual score, threshold quantile q=0.95, and BWS definition, except where a controlled comparison is stated. Root mean square error (RMSE) and mean absolute error (MAE) are computed after inverse transformation in the native numerical units of the released vibration field; the public release does not declare an SI unit for this column, so no unsupported physical-unit conversion is applied. The mean absolute percentage error (MAPE) is computed after inverse transformation and reported as a percentage. Warning results measure rapid-change proxy events; verified fault assessment requires maintenance evidence.

For each horizon, the empirical quantile of validation residual scores determines the warning threshold. The threshold remains fixed during test evaluation, and validation calibration records are excluded from the reported test metrics. An alert-positive record is an evaluated record assigned a positive final alert indicator; a warning episode is a contiguous run of alert-positive records. Alert coverage is the fraction of evaluated records that are alert-positive, with a 2.00% confirmation coverage budget. Residuals and temporal warning operations are defined within each horizon–seed stream; the reported metrics summarize these separate evaluations. For H=1/12/24/48, each seed has 4799/4788/4776/4752 test evaluation records. The integer alert limit is $\lfloor 0.02N_{H}\rfloor =95$ per horizon–seed stream, where $N_{H}$ is the corresponding test record count. The corresponding coverage ceilings are 1.9796%, 1.9841%, 1.9891%, and 1.9992%. HydroPrior retains 95 alert-positive records in each horizon–seed stream, giving mean realized coverage of 1.9880%, reported as 0.0199. Other confirmed models may retain fewer records when candidate availability and minimum spacing limit budget use.

The comparison covers distinct forecasting families. Persistence and simple exponential smoothing (SES) are statistical references; LSTM is recurrent; DLinear is decomposition-linear; and PatchTST, TimesNet, iTransformer, Autoformer, and Informer represent patch-based, temporal-variation, inverted-token, decomposition-attention, and efficient-attention designs. Autoformer combines decomposition with an Auto-Correlation mechanism [8], while Informer uses ProbSparse attention for efficient long-sequence forecasting [7]. Both serve as established architectural references alongside the competitive PatchTST and iTransformer baselines. Retaining their results provides a broader account of model behavior under the evaluated hydropower conditions; the observed ranking describes performance within this setting. In every result table, HydroPrior (ours) denotes the complete framework with a DLinear backbone, the target and context channels, five frozen LLM-prior features, the bounded two-layer correction head described below, and the adaptive confirmation layer for warning metrics.

Table 2 summarizes the dataset and training protocol.

The matched prior-generation comparison includes four configurations: No-prior, Statistical-prior, ML-prior, and LLM-prior. All four share the same five non-target channels, original inputs, DLinear backbone, correction-head architecture, chronological splits, three seeds, and forecast horizons. No-prior disables the five prior-feature inputs while retaining the correction interface. Statistical-prior supplies five window descriptors: window mean, window standard deviation, first-order change, short-window linear trend, and a cross-channel correlation statistic. Its fitted scaling and statistical parameters are estimated on the training split and then frozen; window descriptors are computed from the available input history. ML-prior uses a lightweight autoencoder to learn a five-dimensional bottleneck representation from the same non-target channels. The autoencoder is trained only on the training split, after which its encoder is frozen. LLM-prior uses the frozen rule-derived features. All four configurations use the same validation-threshold calibration, q=0.95, confirmation layer, BWS weights, and 2% coverage budget. Only the prior-feature generation strategy varies.

Table 3 summarizes the warning and correction-head settings. The common warning configuration uses (5), the calibration-window threshold in (6), and q=0.95. HydroPrior sets $\lambda_{c}=20$, κ=2.00%, $\Delta_{\mathrm{alert}}=6$, and ϵ=0. These values control context-evidence weighting, confirmed coverage, minimum alert spacing, and the audit tail. BWS uses fixed budgets of $f_{0}=5$ false alarms per 1000 non-transition points and $\rho_{0}=0.02$ alert coverage. They represent limits on routine false-alarm checks and the fraction of the timeline requiring operator review. BWS assigns weights of 0.45, 0.35, and 0.20 to transition coverage, false-alarm-budget compliance, and alert-coverage-budget compliance, respectively. Event coverage receives the largest individual weight, while the two burden-related terms jointly receive 0.55. These weights express the evaluation preference adopted in this study. The three underlying indicators are reported separately.

Forecasting metrics measure signal tracking at each horizon. Warning metrics measure the corresponding residuals after the common score, threshold, and alert-processing rules are applied. Reporting both stages separates prediction accuracy from the operational burden of the resulting alerts.

The released table has no verified point-level fault labels. Rapid signal transitions are therefore used as reproducible proxy events. They indicate abrupt vibration changes; fault attribution requires future validation against maintenance logs or operator-reviewed intervals.

The forecasting tables report accuracy across model families and horizons. The warning tables report BWS together with transition hit rate, false alarms, and alert coverage. Threshold-sensitivity figures show how BWS and hit rate change with *q*. These views jointly describe prediction quality and warning burden.

For learned models, each horizon mean and sample standard deviation uses three seeds. Avg. is the arithmetic mean of the four horizon means; its accompanying dispersion is the arithmetic mean of the four horizon-specific sample standard deviations. This summary describes seed variability across horizons. The deterministic baseline variability is n/a. Table 4 specifies the offline prior-generation protocol.

### 4.2. Forecasting Evidence

Table 5 shows that HydroPrior has the lowest average RMSE, MAE, and MAPE, as well as the lowest H=48 RMSE. Its closest competitors are iTransformer, PatchTST, DLinear, TimesNet, and LSTM. The horizon-wise results in Table 6 confirm that HydroPrior has the lowest RMSE at H=1, 12, 24, and 48.

Table 7a compares four prior-generation strategies within the correction framework. LLM-prior obtains the lowest mean RMSE at all four horizons, followed by ML-prior, Statistical-prior, and No-prior. Relative to No-prior, the reported RMSE reductions are 4.43%, 3.41%, 2.63%, and 1.10% at horizons 1, 12, 24, and 48, respectively. The corresponding reductions relative to ML-prior are approximately 2.10%, 1.41%, 0.97%, and 0.39%. The comparison supports the value of the frozen LLM features within the evaluated feature-generation strategies, with the smallest difference at the longest horizon.

The horizon-wise gains support the forecasting benefit of frozen operating priors under the evaluated protocol. The improvement decreases with the horizon, reaching 1.10% relative to No-prior at H=48.

Short horizons mainly test local tracking, where current operating context is most informative. Longer horizons amplify smoothing bias, operating-regime drift, and accumulated error. Warning behavior also depends on when residual peaks occur, so the next analysis evaluates the alert stream directly.

These forecasting results establish the quality of the residual source. The warning results below determine whether those residuals can be converted into sparse and useful alerts.

### 4.3. Warning Evidence

Table 8 reports warning performance under the proxy-event protocol. HydroPrior achieves the highest BWS, 0.7492±0.0405, with a transition-hit rate of 0.9106, 0.15 false alarms per 1000 non-transition points, and 0.0199 alert coverage. Several residual baselines reach higher hit rates by flagging much larger portions of the timeline. The raw indicators therefore show that HydroPrior’s BWS advantage comes mainly from controlling false alarms and alert coverage while retaining high transition coverage.

For example, DLinear reaches a hit rate of 0.9866, while its alert coverage rises to 0.2596 and its false-alarm density to 162.39. HydroPrior concentrates its alerts within the 2% budget and keeps false-alarm density close to zero. This comparison illustrates why transition hit rate, false-alarm density, and alert coverage must be interpreted together.

Table 9 applies the same confirmation mechanism, q=0.95, and 2% coverage budget to HydroPrior, DLinear, LSTM, and PatchTST. HydroPrior retains the highest BWS and hit rate and the lowest measured false-alarm density. Because confirmation conditions are matched, the observed difference is consistent with the forecasting results reported in Table 5, Table 6 and Table 7.

**Figure 2:**
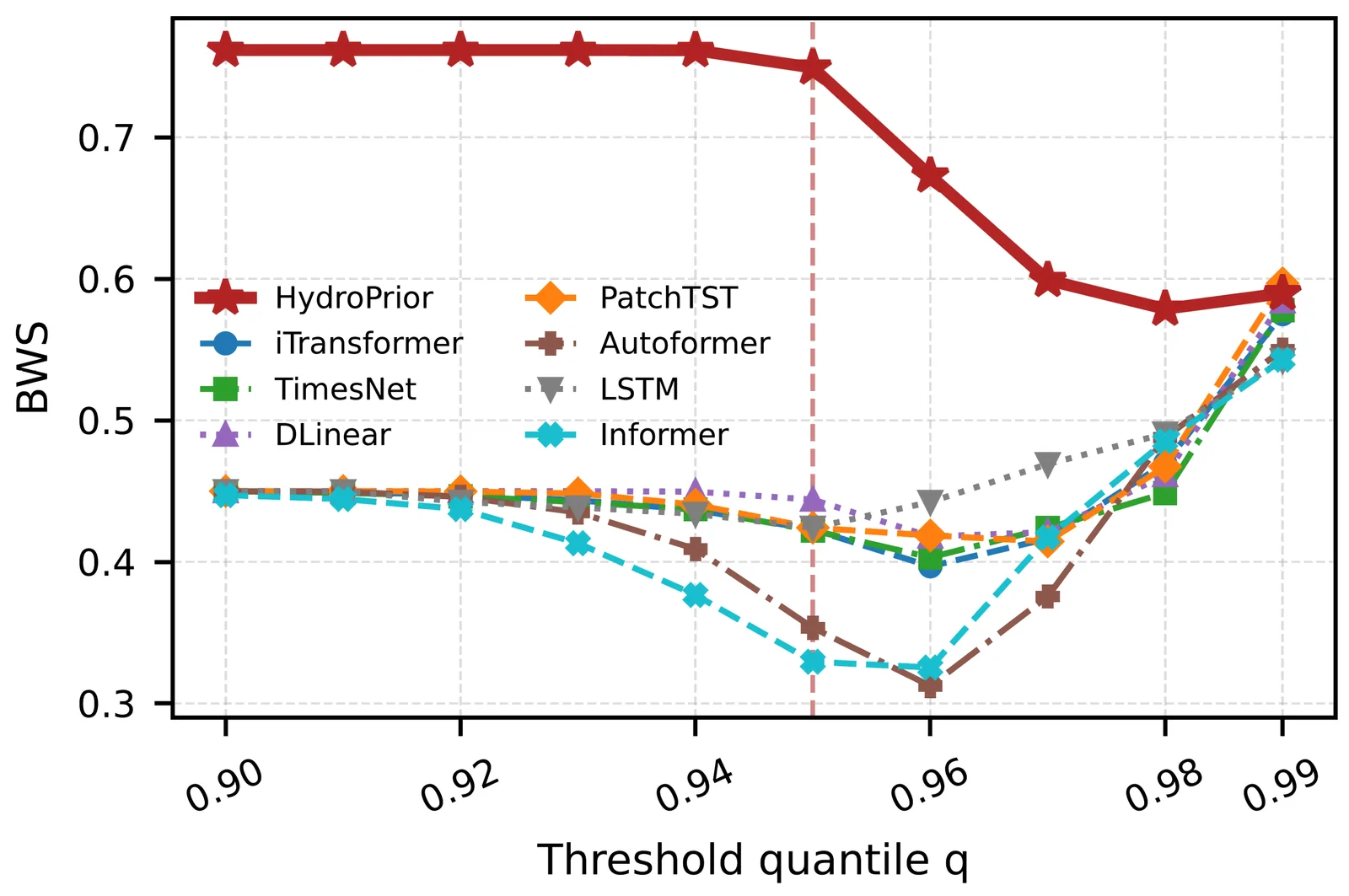
BWS sensitivity of the hydropower warning protocol under a threshold-quantile sweep. The red dashed line marks the common candidate-alert quantile, q=0.95; higher BWS is better.

**Figure 3:**
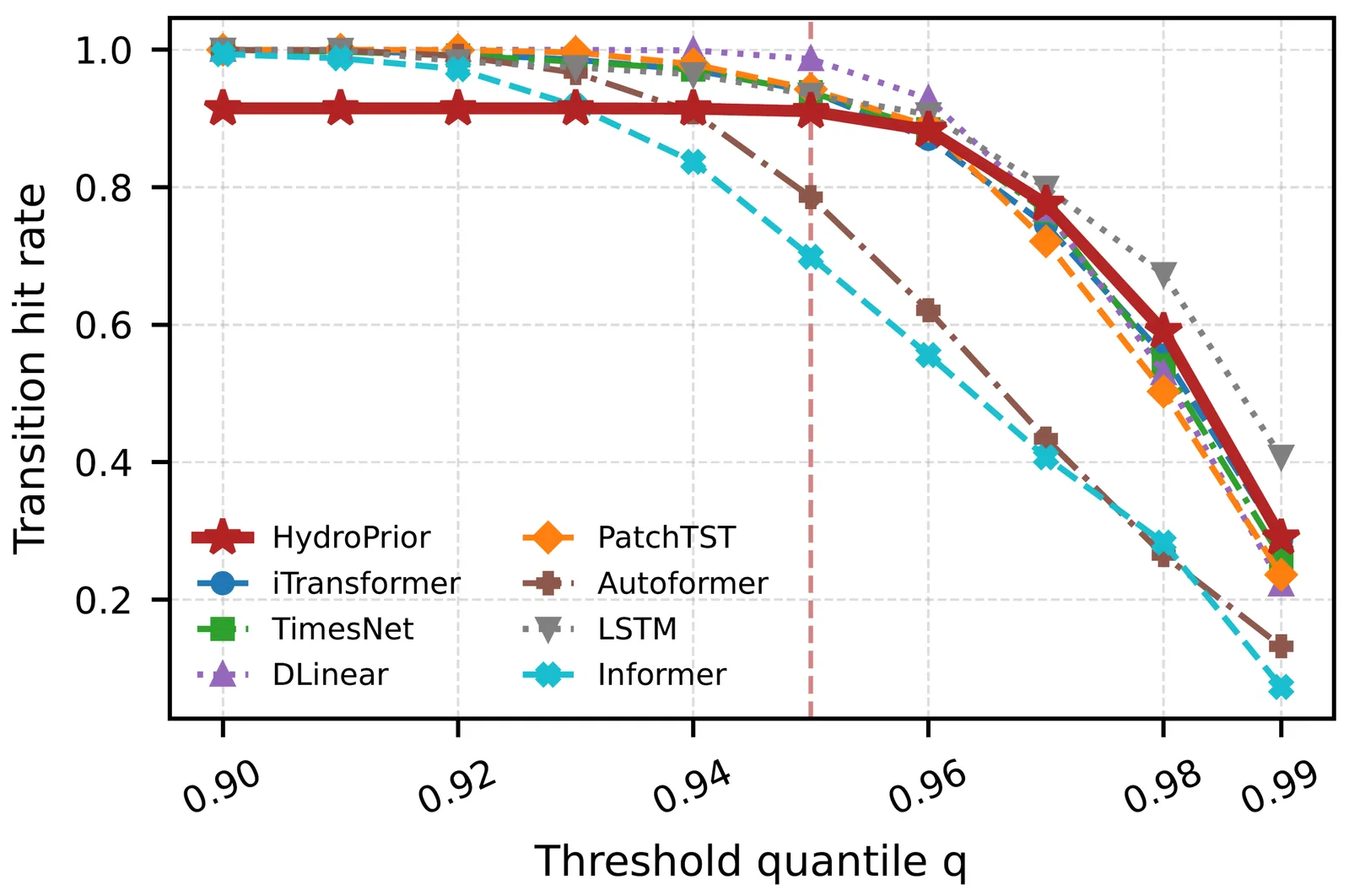
Transition-hit sensitivity under a uniform threshold-quantile sweep over q=0.90–0.99 with step size 0.01. The curve should be interpreted together with the false-alarm and alert-coverage indicators in Table 8.

Figure 2 varies *q* from 0.90 to 0.99 in steps of 0.01; the red dashed line marks the common setting q=0.95. HydroPrior has the highest BWS from q=0.90 through 0.98. At q=0.99, a residual-only baseline becomes competitive because alerts are extremely sparse. Figure 3 shows the corresponding hit rates. Lower thresholds increase transition coverage for the residual baselines, together with a larger alert burden.

Across the common-threshold and equal-budget tests, HydroPrior covers most rapid-transition proxy events while limiting the intervals that require review. The resulting sparsity is consistent with the deployment objective of bounded operator attention on the evaluated single-unit record. Operational suitability requires operator review, event grouping, and validation across seasonal and load-regime changes.

### 4.4. Prior-Feature Ablation and Interpretation

The LLM is used once before training. It organizes channel semantics and training-set statistics into frozen rules for load, thermal behavior, vibration coupling, and operating change. Deterministic transformations convert these rules into five numerical features, which enter the residual-correction head described in Section 3.

The frozen rule file makes each feature traceable and keeps routine warning operation deterministic. Table 7 compares the feature-generation strategies at both the forecasting and warning stages. Under the common 2% coverage budget, LLM-prior attains a transition-hit rate of 0.9106 and a false-alarm density of 0.15, compared with 0.9023 and 0.28 for ML-prior, 0.8921 and 0.39 for Statistical-prior, and 0.8734 and 0.62 for No-prior. These mean differences favor LLM-prior within the tested alternatives. The ordering provides descriptive evidence, with the reported dispersion indicating the variability of the estimates. Table 8 places the complete HydroPrior warning stream in the broader model comparison.

The RMSE reduction decreases from 4.43% at H=1 to 1.10% at H=48. This pattern suggests that frozen operating context is most useful for near-term behavior and has less influence as forecast uncertainty accumulates. The compact correction path and frozen rules support the feasibility of deterministic execution; a complete deployment claim also requires measured latency, memory, and cross-unit validation.

The warning results apply to rapid-change proxy events in the evaluated hydropower data. Maintenance logs and operator-reviewed event intervals are needed to determine fault-level accuracy. They also provide a natural basis for future event-level validation and threshold adaptation across seasons and load regimes.

## 5. Conclusions

This paper presents HydroPrior for residual-based hydropower bearing vibration warning when verified fault labels are scarce. An offline LLM converts channel semantics and training-set statistics into frozen operating priors, while a lightweight forecaster and confirmation layer produce the warning stream. HydroPrior obtains the lowest average forecasting error across the tested statistical, recurrent, decomposition-linear, patch-based, and Transformer-family models. Under the proposed proxy-warning protocol, it achieves the highest BWS, with a transition-hit rate of 0.9106, 0.15 false alarms per 1000 non-transition points, and 0.0199 alert coverage. It also remains ahead of DLinear, PatchTST, and LSTM when all four models use the same confirmation mechanism and coverage budget.

The results support the feasibility of evaluating both signal prediction and alert behavior in label-scarce monitoring on the studied unit. The current evidence concerns one six-month record and rapid-change proxy events. Chronological testing measures performance on later records from the same unit; cross-unit and cross-plant generalization have yet to be established. Validation against maintenance logs, operator-reviewed intervals, additional seasons, operating conditions, and other units is the next step toward fault-level and cross-plant assessment.

## Figures and Tables

**Table 1:** Core notation.

Symbol	Definition
*t*	Forecast origin at the end of the input window
*h*	Forecast horizon, h∈{1,12,24,48}
i=t+h	Timestamp at which the target is realized
$h_{\mathrm{ctx}}$	Fixed-length context summary for correction
$z_{i}$	Confirmation score for candidate alert *i*

**Table 2:** Hydropower data and training protocol.

Item	Protocol Value
Sampling and span	Released 10 min records from 2020-06-01 00:00 to 2020-11-28 18:30
Sample size	23,997 complete records; 12 numerical monitoring channels plus timestamp
Target channel	Upper-guide-bearing vertical vibration
Context channels	Power output, unit speed, bearing-pad temperatures, and guide-bearing vibration channels
Training split	2020-06-01 00:00 to 2020-10-06 13:00; 16,797 rows; 16,701/16,690/16,678/16,654 windows for H=1/12/24/48
Validation split	2020-10-06 13:10 to 2020-10-23 05:10; 2401 rows; 2401/2390/2378/2354 windows
Test split	2020-10-23 05:20 to 2020-11-28 18:30; 4799 rows; 4799/4788/4776/4752 windows
Leakage control	Chronological 70/10/20 split; each later split receives 96 preceding rows as input context only; normalization and prior statistics are fitted on training rows
Window and horizons	Input window L=96; horizons H∈{1,12,24,48}
Warning calibration	Validation residual scores; horizon-specific thresholds fixed before test evaluation
Warning evaluation	Test records only; excludes validation calibration records; common 2.00% coverage budget; integer upper bound of 95 records per horizon–seed stream
Training protocol	Three seeds, batch size 64, maximum 20 epochs, patience 5
Timestamp validation	No invalid or duplicate timestamps; five non-10 min acquisition gaps; 2035 missing grid slots were not interpolated
Data quality	No missing sensor values observed in the retained complete records

**Table 3:** Warning conversion and HydroPrior correction-head configuration.

Component	Fixed Setting	Purpose
Residual score	a=0.5, b=0.3, c=0.2, rolling window w=12	Combine residual level, residual change, and local volatility
Threshold	Validation-score quantile with q=0.95 and horizon-specific $W_{\tau }^{\left(h\right)}$	Convert processed residual scores into candidate warnings
Confirmation	$\lambda_{c}=20$, κ=2.00%, $\Delta_{\mathrm{alert}}=6$, ϵ=0	Reallocate candidates and enforce 60 min spacing within contiguous segments
BWS budgets	$f_{0}=5$ FA/1k, $\rho_{0}=0.02$ alert coverage	Reward compliance with fixed false-alarm and timeline-coverage budgets
Correction head	Two-layer MLP, hidden width 128, Gaussian error linear unit (GELU), dropout 0.05, correction bound $0.4\sigma_{y}$	Inject frozen LLM-prior features while limiting overcorrection
Optimization	Joint mean squared error (MSE) training with the selected lightweight forecaster; no proxy labels used for tuning	Preserve forecasting supervision and keep warning evaluation separate

**Table 4:** Reproducible offline LLM-prior generation protocol.

Item	Fixed Setting	Prior-Rule Example
LLM backend	Offline LLM backend implemented with Qwen2.5-7B-Instruct, temperature 0.0, top-p=1.0, strict JavaScript Object Notation (JSON) output, and three deterministic format checks.	The frozen JSON rule artifact, prompt template, model identifier, rule-format version, and creation date are treated as the reproducible objects.
Prompt template	System block: hydropower condition-monitoring assistant. User block: channel list, physical semantics, training-segment statistics, and requested rule families. Output block: structured rule file with no free-form prose.	Rule families include load context, thermal context, vibration coupling, transition risk, and regime-change tendency.
Input statistics	Training segment statistics include missing rate, mean, standard deviation, min/max, 0.05/0.25/0.50/0.75/0.95 quantiles, and first-difference quantiles for every available channel.	Training-only first-difference quantiles form prior features; evaluation transition thresholds are computed separately for proxy-event analysis and are not used in training or alert generation.
Freezing rule	The validated JSON is saved before model fitting. All downstream data-preparation scripts read this file and reuse the normalization constants, quantile thresholds, and rule weights.	Validation and test segments use the same frozen transformations as the training-derived prior generator.
Consistency check	Deterministic decoding is used first; if backend nondeterminism changes rule fields, repeated generation must return identical rule-family names, channel coverage, and transformation types before freezing.	The frozen rule file is consumed unchanged by forecasting, residual correction, and warning evaluation.
Rule example	Load and thermal rules summarize operating-condition intensity; vibration-coupling rules summarize cross-channel vibration agreement; transition rules summarize rapid-change tendency.	The transition-risk feature increases when non-target vibration coupling and operating-context variation jointly enter high training-segment quantile regions.

**Table 5:** Hydropower forecasting performance on the inverse-transformed dataset-native scale. Avg. denotes the horizon average; lower values are better.

Model	Avg. RMSE	Avg. MAE	MAPE (%)	H=48 RMSE	Horizons	Runs
**HydroPrior (Ours)**	0.4382±0.0021	0.2842±0.0007	1.1226±0.0072	0.5048±0.0047	4	12
iTransformer	0.4501±0.0027	0.2865±0.0016	1.1792±0.0076	0.5239±0.0047	4	12
PatchTST	0.4506±0.0044	0.2883±0.0027	1.1614±0.0134	0.5245±0.0118	4	12
DLinear	0.4507±0.0050	0.2932±0.0038	1.1443±0.0169	0.5104±0.0047	4	12
TimesNet	0.4542±0.0080	0.2900±0.0035	1.1779±0.0182	0.5264±0.0102	4	12
LSTM	0.4569±0.0077	0.3034±0.0020	1.1666±0.0185	0.5457±0.0179	4	12
Autoformer	0.5202±0.0029	0.3408±0.0026	1.3753±0.0146	0.5653±0.0020	4	12
Informer	0.5867±0.0320	0.4427±0.0340	2.4542±0.3135	0.6703±0.0628	4	12
SES	2.8842 (n/a)	1.8418 (n/a)	10.4501 (n/a)	3.3361 (n/a)	4	4
Persistence	3.5245 (n/a)	2.2796 (n/a)	12.8075 (n/a)	3.9188 (n/a)	4	4

Bold numerical entries indicate the lowest error in each column; bold model names identify HydroPrior. n/a denotes seed variability that is not applicable to deterministic models.

**Table 6:** Horizon-wise hydropower RMSE on the inverse-transformed dataset-native scale. Learned-model entries are the mean ± sample standard deviation over three seeds. Avg. follows the definition in the experimental settings; the deterministic baseline variability is n/a. Lower values are better.

Model	H=1	H=12	H=24	H=48	Avg.
**HydroPrior**	0.3778±0.0003	0.4186±0.0029	0.4515±0.0006	0.5048±0.0047	0.4382±0.0021
iTransformer	0.3813±0.0019	0.4274±0.0027	0.4680±0.0015	0.5239±0.0047	0.4501±0.0027
PatchTST	0.3935±0.0005	0.4242±0.0036	0.4601±0.0018	0.5245±0.0118	0.4506±0.0044
DLinear	0.3953±0.0049	0.4334±0.0058	0.4637±0.0046	0.5104±0.0047	0.4507±0.0050
TimesNet	0.3912±0.0084	0.4328±0.0091	0.4665±0.0044	0.5264±0.0102	0.4542±0.0080
LSTM	0.3802±0.0002	0.4232±0.0019	0.4787±0.0107	0.5457±0.0179	0.4569±0.0077
Autoformer	0.4673±0.0038	0.5155±0.0028	0.5328±0.0032	0.5653±0.0020	0.5202±0.0029
Informer	0.4608±0.0206	0.5802±0.0274	0.6353±0.0174	0.6703±0.0628	0.5867±0.0320
SES	2.4814 (n/a)	2.7066 (n/a)	3.0126 (n/a)	3.3361 (n/a)	2.8842 (n/a)
Persistence	3.2000 (n/a)	3.3585 (n/a)	3.6218 (n/a)	3.9188 (n/a)	3.5245 (n/a)

Bold numerical entries indicate the lowest RMSE in each column; bold model names identify HydroPrior. n/a denotes seed variability that is not applicable to deterministic models.

**Table 7:** Matched comparison of prior-generation strategies. Panel (**a**) reports RMSE mean ± sample standard deviation over three seeds on the inverse-transformed dataset-native scale; lower is better. Panel (**b**) reports transition-hit mean and standard deviation, mean false-alarm density, and the configured coverage budget under a common confirmation protocol; higher hit and lower false-alarm density are better. LLM-prior is the HydroPrior configuration.

**(a) Forecasting Performance**				
**Horizon**	**No-Prior**	**Statistical-Prior**	**ML-Prior**	**LLM-Prior**
1	0.3953±0.0017	0.3918±0.0015	0.3859±0.0018	0.3778±0.0016
12	0.4334±0.0021	0.4298±0.0019	0.4246±0.0020	0.4186±0.0018
24	0.4637±0.0024	0.4602±0.0023	0.4559±0.0022	0.4515±0.0021
48	0.5104±0.0028	0.5088±0.0027	0.5068±0.0026	0.5048±0.0025
**(b) Warning Performance**				
**Feature Configuration**		**Transition Hit**	**FA/1k**	**Coverage Budget**
No-prior + confirmation		0.8734±0.0418	0.62	2.00%
Statistical-prior + confirmation		0.8921±0.0416	0.39	2.00%
ML-prior + confirmation		0.9023±0.0416	0.28	2.00%
LLM-prior + confirmation		0.9106±0.0405	**0.15**	2.00%

Bold numerical entries indicate the lowest RMSE for each horizon, the highest transition hit, or the lowest false-alarm density in the matched comparison. Coverage budget is the configured upper limit.

**Table 8:** Hydropower proxy-warning comparison with raw warning indicators under the common warning protocol. HydroPrior (Ours) uses the same DLinear-backed prior-corrected forecaster as Table 5 and enables its adaptive confirmation layer; residual baselines use the shared residual-threshold protocol. BWS uses fixed budgets $f_{0}=5$ false alarms per 1000 non-transition points and $\rho_{0}=0.02$ alert coverage; Records denotes horizon–seed records.

Model	BWS	Hit	Alert Cov.	FA/1k	Records
**HydroPrior**	**0.7492 ± 0.0405**	0.9106	0.0199	**0.15**	12
DLinear	0.4440±0.0119	**0.9866**	0.2596	162.39	12
PatchTST	0.4242±0.0541	0.9426	0.1968	112.65	12
LSTM	0.4241±0.0596	0.9344	0.2270	135.56	12
iTransformer	0.4229±0.0371	0.9397	0.1657	89.96	12
TimesNet	0.4222±0.0517	0.9383	0.1694	92.81	12
Autoformer	0.3535±0.0542	0.7855	0.0593	26.34	12
Informer	0.3295±0.1003	0.6988	0.0821	44.40	12

Bold numerical entries indicate the highest BWS, the highest hit rate, and the lowest false-alarm density; bold model names identify HydroPrior. Alert coverage is reported as a fraction.

**Table 9:** Confirmation-layer comparison under a common coverage budget. All rows use q=0.95 and a 2% confirmation coverage budget; Records denotes horizon–seed records.

Variant	BWS	Hit	Alert Cov.	FA/1k	Records
HydroPrior (Ours)	0.7492±0.0405	0.9106	0.0199	**0.15**	12
DLinear + confirmation	0.7146±0.0639	0.8699	≤0.0199	0.38	12
LSTM + confirmation	0.7082±0.0858	0.8323	0.0190	0.37	12
PatchTST + confirmation	0.6674±0.1478	0.8421	0.0197	0.92	12

Bold numerical entries indicate the highest BWS and lowest false-alarm density. The coverage entry marked ≤ gives the rounded mean upper bound imposed by the integer budget.

## Data Availability

The source hydropower vibration data analyzed in this study are publicly available on Figshare at https://doi.org/10.6084/m9.figshare.21290895. The processed datasets and experimental outputs generated in this study are not readily available because they are part of an ongoing study. Requests for access should be directed to the corresponding author and are subject to the required authorization. Public release of the custom implementation has not been authorized; the code is currently unavailable for public distribution.

## Abbreviations

The following abbreviations are used in this manuscript:

BWS	Balanced Warning Score
LLM	Large language model
MAPE	Mean absolute percentage error
MSE	Mean squared error
RMSE	Root mean square error
SCADA	Supervisory control and data acquisition

## Appendix A. Frozen LLM-Prior Rule Summary

The frozen rule artifact used by HydroPrior is stored as a JSON file before model fitting and is reused unchanged in data preparation, residual correction, and warning evaluation. The artifact records the LLM backend, deterministic decoding settings, target channel, feature dimension, channel groups, transformation type, rule weight, and training-only statistics. Table A1 lists the rule summary used in the reported experiments.

**Table A1:** Frozen LLM-prior rule summary. Each feature is one-dimensional; all scaling constants, quantiles, and robust ranges are fitted on the training split only.

Feature	Source and Transformation	Weight and Training Statistics
Load	Generator output and unit speed; robust normalized mean	1.00; median, interquartile range (IQR), 0.05/0.95 quantiles
Thermal	Thrust and guide-bearing pad temperatures; robust normalized mean	0.80; median, interquartile range (IQR), 0.05/0.95 quantiles
UGB lateral	upper guide bearing (UGB) horizontal vibration +X/−Y; cross-channel robust contrast	0.90; median, interquartile range (IQR), 0.95 quantile
Cross-bearing	lower guide bearing (LGB) +X/−Y and turbine guide bearing (TGB) +X vibration; cross-channel robust contrast	0.90; median, interquartile range (IQR), 0.95 quantile
Operating change	Load, thermal, and companion-vibration groups; high-quantile variation indicator	1.10; first-difference 0.75/0.95 quantiles

The coefficients in Table A1 were generated by Qwen2.5-7B-Instruct during offline prior construction from channel semantics and training-set statistics, then recorded in the rule file and frozen. They are LLM-proposed heuristic scaling factors. Their generation involves no gradient-based optimization, grid search, validation-set selection, or test-set tuning. Relative to the unit load coefficient, 0.80 attenuates thermal context, 0.90 attenuates each vibration-coupling feature, and 1.10 emphasizes operating change after the feature transformations. Their exact values are taken from the frozen LLM output. Subsequent forecast training updates the forecaster and correction head while keeping the rule coefficients fixed. The comparisons evaluate the complete prior-generation strategy; the contribution of individual coefficient values remains a subject for further controlled evaluation.

The JSON schema contains the following required fields: artifact name, LLM backend, target channel, feature dimension, channel groups, feature name, feature group, feature dimension, feature weight, transformation type, required statistics, and textual description. The authors retain the frozen rule artifact, prompt template, generation settings, and file hash as verification records; these materials are available from the corresponding author upon reasonable request.
